# Beliefs about Pain Control in Patients after Abdominal Aortic Aneurysm Surgery—A Preliminary Study

**DOI:** 10.3390/ijerph19063708

**Published:** 2022-03-20

**Authors:** Renata Piotrkowska, Natalia Sanecka, Wioletta Mędrzycka-Dąbrowska, Piotr Jarzynkowski

**Affiliations:** 1 Department of Surgical Nursing, Medical University of Gdansk, Debinki 7, 80-211 Gdańsk, Poland; p.jarzynkowski@gumed.edu.pl; 2 Clinic of Cardiac and Vascular Surgery, University Clinical Centre, 80-952 Gdańsk, Poland; nataliasanecka@gumed.edu.pl; 3 Department of Anaesthesiology Nursing and Intensive Care, Medical University of Gdańsk, Dębinki 7, 80-211 Gdańsk, Poland; wioletta.medrzycka-dabrowska@gumed.edu.pl

**Keywords:** abdominal aortic aneurysm, postoperative pain, pain control

## Abstract

Introduction: Pain-control beliefs significantly influence the perception of disease and, therefore, may influence the treatment outcomes of surgical patients. The sense of control is related to the sense of agency and the ability to influence one’s own life and environment. This construct may be external or internal. The belief that pain control depends on internal or external factors can depend on many variables. This may be influenced by socio-demographic and clinical characteristics, as well as the source and cause of pain. The aim of the study was the assessment of the relationship between the intensity of postoperative pain and beliefs about pain control in patients after AAA surgery and assessment of the relationship between socio-demographic and clinical variables and beliefs about pain control in patients after AAA surgery. Materials and Methods: The research material consisted of 42 patients aged 57 to 85, hospitalized at the Department of Cardiac Surgery and Vascular Surgery of the University Clinical Center in Gdańsk. The research was conducted from March to September 2020. The study uses a survey technique based on a standardized research tool: the Polish version of the BPCQ (The Beliefs about Pain Control Questionnaire), the NRS (Numerical Rating Scale), and the author’s own questionnaire that allows for the collection of socio-demographic data. Results: The highest intensity of pain was observed in subjects with ruptured AAA H (2) = 6.19; *p* < 0.05 and subjects who underwent classic surgery Z = −2.95; *p* < 0.05 (non-parametric Mann–Whitney U test). Subjects with ruptured aneurysms are less convinced about the influence of internal factors on pain control H (2) = 5.26; *p* < 0.05. The respondents’ conviction about the influence of doctors on pain control increased together with their age, rHO = 0.38, *p* < 0.05. Conclusion: Pain intensity after surgery did not significantly correlate with beliefs about pain control. Patients with ruptured AAA are less convinced about the influence of internal factors on pain control. With age, patients have more confidence in their doctors than in themselves to control their pain.

## 1. Introduction

### 1.1. Abdominal Aortic Aneurysm and Pain

The International Association for the Study of Pain (IASP) updated the 1979 definition of pain, which now reads: “pain is an unpleasant sensory and emotional experience related or similar to, actual or potential tissue damage” [1]. However, it should be taken into account that pain is a perception that arises as a result of the psychological interpretation of the patient. It concerns the phenomena occurring in the patient’s body, which are influenced by previous experiences and psychosomatic conditions. Pain may be a consequence of the irritation of nociceptors or a reduction in their excitability threshold. Pain also arises as a result of damage to the structures of the nervous system or on a psychogenic basis [2].

Abdominal aortic aneurysm (AAA) is a common, asymptomatic disease involving local expansion of the aorta by more than 50% of its normal diameter. Although some patients with AAA experience back or abdominal pain, most remain asymptomatic until rupture. Its severity can vary from mild to strong [3]. The appearance of pain with these features in a person diagnosed with an abdominal aortic aneurysm; its worsening; or radiation into the groin, buttocks, or thighs is particularly disturbing, as this may indicate the imminent rupture of the aneurysm. Progressive dilatation of the artery causes the aneurysm to rupture, killing the patient in 85% of cases. The factors predisposing to the formation of AAA are genetics-related. It is also indicated that an aneurysm is more likely to develop in smokers, overweight or obese people, or after myocardial infarction [4,5]. Epidemiological studies indicate that AAA occurs in 4–7% of men over 50 years of age and in 1–2% of women over 55 years of age [6,7,8].

The main goal of treating an abdominal aortic aneurysm is to prevent the often fatal complication of aneurysm rupture. The only treatments for AAA are classic (open method) or endovascular surgery. The indication for surgery is an aneurysm larger than 55 mm and symptomatic or ruptured aneurysm. The classic method involves implanting a vascular prosthesis into the retroperitoneal space. The endovascular method is the placement of a stent graft within the lumen of the aneurysm through the peripheral vessel [9].

Open abdominal aortic repair usually requires standard median laparotomy. This classic method of removing an abdominal aortic aneurysm is associated with a heavy burden on the patient. Long-term treatment leads to a significant cooling of the body. After surgery, the patient requires observation in the Intensive Care Unit. The extent of the procedure is conducive to the appearance of severe postoperative pain, and to reduce these ailments to a minimum, epidural analgesia is used [9,10,11].

The endovascular procedure involves small incisions in the groin. The patient requires increased observation and care for 24 h, with the difference that the epidural anesthesia used minimizes the risk of respiratory failure. In the following days, the analgesic treatment is limited to mild non-opioid analgesics used according to medical orders [9,10].

### 1.2. Pain-Control Beliefs

Various factors influence the size and extent of postoperative pain. Attention is paid to the influence of non-physical factors that are an important element of the perception of the pain phenomenon. Psychological factors include beliefs about pain control and the ability to use techniques that reduce pain perception [12]. The sense of control is related to the sense of agency and the ability to influence one’s own life and environment. This construct may be external or internal. People with an external sense of control tend to succumb to external influences and pressures; they are convinced that they have no influence on situations and events and feel helpless, depressed, and potentially suicidal [13,14]. They treat illness as a misfortune. This perception of pain leads to a passive attitude. Such an attitude has a negative impact on effective pain control and, as a result, increases the intensity of the perceived psychological discomfort. People who show an internal sense of control are convinced that their lives depend on them; they take responsibility for their own actions and decisions. This belief reduces the level of perceived pain-related stress and increases pain tolerance. In these patients, there is a tendency to better cope with pain and to cooperate better with medical staff [13,14].

### 1.3. Aim

The aim of the study was the assessment of the relationship between the intensity of postoperative pain and beliefs about pain control in patients after AAA surgery and the assessment of the relationship between socio-demographic and clinical variables and beliefs about pain control in patients after AAA surgery.

## 2. Materials and Methods

This study had a descriptive and relevant research design using the prospective collection of data from the medical records of patients hospitalized in a hospital in northern Poland.

Data collected from the medical records of hospitalized patients included age, sex, disease category, admission date, discharge date, hospitalization days, and pain level. Pain scores in this study were obtained from patients who self-rated their pain scores on a 0–10 point numerical scale. Pain-control beliefs were obtained from patients based on the BPCQ scale.

### 2.1. Design

This was a cross-sectional survey study. The STROBE checklist was used to ensure quality reporting during the study.

### 2.2. Study Procedures

The research was carried out at the Department of Cardiac Surgery and Vascular Surgery of the University Clinical Center in Gdańsk in 2020. The material consisted of 42 patients aged 57 to 85 years who underwent AAA (open surgery or endovascular aneurysm repair—(EVAR)). The inclusion criteria included informed consent form, a diagnosis of AAA, and age > 18 years. We excluded patients who did not consent or submitted incomplete questionnaires. The respondents were subjected to the study with two time assessments: on day 0 and the second day after surgery.

The qualification for surgical treatment of an abdominal aortic aneurysm was in line with the Polish Recommendations for the Endovascular Treatment of Peripheral Artery and Aortic Diseases 2009 [10]:

- Patients who did not meet anatomical criteria for stent graft implantation were qualified for vascular prosthesis implantation, with cardiological and anesthesiological qualifications for general anesthesia,
- Patients who met anatomical criteria and were disqualified from general anesthesia were qualified for stent graft implantation.

### 2.3. Questionnaire Development

An author’s questionnaire was used to collect socio-demographic data. The second research tool was the BPCQ (The Beliefs about Pain Control Questionnaire) scale by S. Skevington (1990) School of Social Sciences, University of Bath, with Polish adaptation by Z. Juczyński and a scale for assessing pain intensity (NRS) [15,16].

#### 2.3.1. BPCQ Scale

This refers to scales measuring the location of control, including health control. This scale assesses the strength of individual beliefs about pain control using internal factors, the influence of doctors (the strength of others), and random events. It is intended to examine adults, the sick, and those who complain of pain. It can also measure beliefs about pain control in people who do not currently complain of pain [15,16].

The respondents assessed the questionnaire’s statements, basing their opinion on the Likert scale ranging from 1 = no, completely disagree to 6 = yes, completely agree.

The BPCQ developed by Skevington consists of 13 items assigned to 3 subscales measuring beliefs about internal (personal control) of pain (IS), beliefs that powerful others (i.e., doctors) can control pain (PD), and beliefs that pain is controlled by chance events (CH). Higher scores in these subscales indicated stronger endorsements of the respective belief. The range of points for the location of internal control is 5–30 points; for the remaining ones, the influence of doctors and chance events is the same and amounts to 4–24 points [14,15]. A higher score indicated a stronger belief of the respondent about the influence of a given factor on pain control. The BPCQ examination was performed on each patient on the second day after the surgery.

#### 2.3.2. NRS

The NRS is a numerical scale with 11 pain levels ranging from 0 to 10, where 0 is no pain at all and 10 is the worst pain imaginable. The NRS score of up to 3 points means mild pain, 4–6 points is moderate pain, and the score 7–10 points is acute pain [17].

The scale was used in each subject twice on the “zero” and “second” days after the surgery.

### 2.4. Ethical Considerations

While collecting the data, the ethical principles set out in the Helsinki Declaration were taken into account. The research was approved by the Independent Bioethical Committee at the Medical University of Gdańsk, number NKBBN/124/2020.

### 2.5. Statistical Analysis

All statistical calculations were performed using the IBM SPSS 23 statistical suite and Excel 2016. Qualitative variables were presented using counts and percentages, and quantitative variables were characterized using an arithmetic mean and standard deviation. The significance of differences between more than two groups was checked with the Kruskal–Wallis test (in the case of obtaining significant differences, Bonferroni post hoc tests were used), and between two groups was checked with the Mann–Whitney U test. To find the relationship of strength and direction between the variables, a correlation analysis was used to calculate the Spearman correlation coefficients. In all calculations, *p* ≤ 0.05 was assumed as the level of significance.

## 3. Results

### 3.1. Socio-Demographic Characteristics of the Study Group

Men accounted for the more numerous group of respondents, 83.3% (*n* = 35), while women accounted for 16.7% (*n* = 7). The age of the respondents ranged from 57 to 85 years of age, M = 70.4. The most numerous group of patients was 64 years old and accounted for 21% (*n* = 9). Among the respondents, the largest number of people were married residents of cities—61.9% (*n* = 26). A total of 33.3% (*n* = 14) had vocational education, 35.7% (*n* = 15) had secondary education, and only 11.9% (*n* = 5) had higher education. A significant part of the respondents, 54.8% (*n* = 23), confirmed that their main source of income was retirement pension. A total of 57.1% (*n* = 24) of patients were diagnosed with asymptomatic abdominal aortic aneurysm, symptomatic AAA was present in 35.7% (*n* = 15), and ruptured AAA was present in 7.1% (*n* = 3). Most of the respondents reported the presence of comorbidities. The study group most often exhibited arterial hypertension (23.5%, *n* = 31), followed by nicotine addiction (20.5%, *n* = 27), heart disease (9.8%, *n* = 13), and diabetes mellitus (8.3%, *n* = 11). The average number of cigarettes smoked per day was M = 14.73, SD = 7.03. Patients after classic abdominal aortic aneurysm surgery (*n* = 28) were more numerous (*n* = 28), and the group of patients who underwent endovascular treatment of abdominal aortic aneurysm (EVAR) 33.3% (*n* = 14) was smaller.

### 3.2. Assessment of Pain Intensity with the NRS

#### 3.2.1. Measurement on Day Zero after Surgery

The examined patients showed pain intensity on day zero after the surgery, on average 3.81 points, SD = 1.97. Pain on day zero was diversified in terms of intensity. The lowest pain intensity at the time of the examination was 0 points, and the highest pain intensity was 8 points on the NRS. The applied non-parametric U Mann–Whitney test showed that gender did not change the level of pain intensity on day zero after surgery Z = −0.47; *p* > 0.05. On the other hand, the Spearman correlation test showed that with the increase in the age of the respondents, the intensity of pain decreased on day zero on the NRS rHO = −0.49, *p* < 0.05. No statistically significant correlation was found between the BMI level of the subjects and the pain level, rHO = 0.05, *p* > 0.05. The subjects who underwent the classic procedure Z = −4.36 had a statistically significantly higher level of pain on day zero; *p* < 0.001 (non-parametric Mann–Whitney U test).

Statistical analysis conducted using the Kruskal–Wallis test showed a relationship between the type of aneurysm and the intensity of pain. Further analysis by means of multiple comparisons showed that the subjects with ruptured AAA had the highest level of pain intensity compared to those with asymptomatic and symptomatic aneurysm, at a level of statistical significance, H (2) = 6.19; *p* < 0.05.

#### 3.2.2. Measurement on the Second Day after Surgery

The examined patients showed pain intensity on the second day after the surgery, on average 1.4 points, SD = 1.64. Gender did not change the level of pain intensity on the second day after surgery, Z = −0.31; *p* > 0.05 (non-parametric Mann–Whitney U test). There was no statistically significant correlation between BMI, rHO = 0.02, *p* > 0.05, and age, rHO = −0.28, *p* > 0.05, and the level of pain on the second day after surgery (Spearman’s correlation test). The subjects who underwent the classic procedure, Z = −2.95, had a statistically significantly higher level of pain on the second day after the procedure; *p* < 0.05 (non-parametric Mann–Whitney U test).

#### 3.2.3. Pain intensity on Day Zero and the Second Day after Surgery

Student’s T-Test was applied. The analysis showed that the pain on day zero was statistically significantly stronger than on the second day after surgery t (41) = 14.09; *p* < 0.001.

### 3.3. Assessment of Beliefs about Pain Contro—BPCQ

The BPCQ results show that patients operated on for AAA believe that internal factors play the greatest role in pain control, with an average of 21.02 and a standard deviation of 4.96 (Table 1).

No statistically significant correlation was found between the intensity of pain and the point values obtained for the individual parameters of pain control on day zero (internal control, rHO = −0.21, *p* > 0.05; physicians’ influence, rHO = 0.06, *p* > 0.05; and the influence of chance, rHO = −0.03, *p* > 0.05) and on the second day after surgery (internal control, rHO = −0.25, *p* > 0.05; doctors’ influence, rHO = 0.12, *p* > 0.05; and the influence of chance, rHO = −0.18, *p* > 0.05).

#### 3.3.1. Internal Control

The gender of respondents (Z = 0.42; *p* > 0.05), age (rHO = −0.04, *p* > 0.05), BMI (rHO = 0.09, *p* > 0.05), and type of surgery (Z = 0.06; *p* > 0.05) did not affect beliefs about pain control in relation to internal control (Table 2). Only the subjects with ruptured aneurysms were less convinced about the influence of internal factors on pain control, H (2) = 5.26; *p* < 0.05 (Table 3). In subjects with concomitant nicotine addiction, the belief about the influence of internal control decreased with the increase in the number of smoked cigarettes, rHO = −0.49, *p* < 0.05 (Table 4).

#### 3.3.2. The Influence of Doctors

Statistical analysis showed that with increasing age, the respondents’ conviction about the influence of doctors on pain management increased, rHO = 0.38, *p* < 0.05. The other socio-demographic and clinical variables did not significantly affect beliefs about pain control (Table 5, Table 6 and Table 7).

#### 3.3.3. Influence of Chance

Pain control through the influence of chance in patients with nicotine addiction was significantly dependent on the number of cigarettes smoked. As the number of cigarettes smoked per day increased, so was the perception that chance could influence pain control. The other socio-demographic and clinical variables did not significantly affect beliefs about pain control. (Table 8, Table 9 and Table 10).

## 4. Discussion

Research on beliefs about pain control is most often conducted among cancer patients [18,19,20,21,22]. Only a few studies on subjective pain perception and beliefs about control and coping with it have been conducted on surgical patients [23,24,25,26,27]. Pain-control beliefs significantly influence the perception of disease and, therefore, may influence the treatment outcomes of surgical patients. Psychological modeling of beliefs about pain control can be a valuable way to improve overall clinical outcomes [28,29]. Cognitive–behavioral interventions regarding beliefs about pain control may be appropriate to improve postoperative functioning [30].

In our study, the BPCQ results show that patients undergoing AAA surgery believe that internal factors play the greatest role in pain control, with an average of 21.02 and a standard deviation of 4.96. In the case of doctors’ influence, the mean score was 19.09, and the standard deviation was 3.90. In turn, the mean result for random events was 15.28, and the standard deviation was 3.69. The inner sense of control is positively correlated with the belief in a strong ability to control pain. Such an attitude may translate into a reduction in the feeling of hopelessness and depression, and it helps to better cope with pain. The belief that pain control depends on internal or external factors can depend on many variables. This may be influenced by socio-demographic and clinical characteristics, as well as the source and cause of pain.

Studies have shown that patients with chronic back pain and multiple sclerosis show greater internal control. Women with breast or uterine cancer, patients with chronic pain, and patients with chronic ischemia of the lower limbs show that physicians have the greatest influence on pain control. In the group of 100 patients with peripheral arterial disease in the study by Kadłubowska et al. [31], the median influence of internal factors was 18 points, the influence of doctors 19 points, and the effect of random events was 16 points. The same study also included 100 patients with rheumatoid arthritis. In this group of patients, all three groups of factors influencing the perception of pain obtained lower scores than in patients with peripheral arterial disease (internal factors, 14 points; doctors, 16 points; chance events, 15 points). The BPCQ results in the study by Kowalczuk et al. showed that the respondents’ perception of pain was average. Most respondents similarly rated the impact of all three pain control agents contained in the BPCQ [27].

Although beliefs about internal control are more strongly associated with better mental and physical health, belief in physician control may be stronger during illness [32].

In our study, internal beliefs about pain control seem to be independent of age, gender, and the type of surgery received.

In the group of patients operated on for AAA, we can see that patients with ruptured aneurysms are less confident about the impact of internal factors on pain control. This confirms the hypothesis that patients treated surgically reveal a stronger belief about the influence of doctors on pain experiences. Severe pain can undermine patients’ confidence in their personal ability to control pain. They may view themselves as being less in control of their pain, and they may view doctors as having more control of their pain. A similar situation is for patients operated on for AAA. Conviction about the role physicians have in controlling pain grows with the age of patients. Nurses and doctors should be aware that their duty is not only to treat the patient but also to provide reliable information about the disease and the causes of pain and to provide psychological support [28,33].

## 5. Conclusions

Pain intensity after surgery did not significantly correlate with beliefs about pain control. Patients with ruptured AAA are less convinced about the influence of internal factors on pain control. With age, patients have more confidence in their doctors than in themselves to control their pain. In patients with nicotine addiction, the belief about the influence of internal factors on pain control decreased, and the belief about the influence of chance significantly increased with the number of cigarettes smoked.

## 6. Study Limitations

A limitation of this study is the low representativeness of the results, which makes it difficult to generalize the obtained results and formulate broader conclusions. The subject of this study was limited to patients hospitalized in one medical center. In a subsequent study, the number of patients tested should be increased. In addition, there are many social, psychological, and clinical variables that can influence the assessment of pain severity and beliefs about pain control, the effects of which have not been taken into account. For example, the effect of pharmacological pain management and levels of anxiety and depression were not accounted for, which can strongly influence beliefs about pain control. We propose to extend the research with additional variables. In the future, further research is needed to further clarify the interactions between the biological and psychosocial processes that have an important impact on pain experience.

## 7. Implications for Practice

Despite the methodological limitations of the study, the obtained results may be useful in understanding the influence of some psychological and clinical factors on pain reporting in patients after abdominal aortic aneurysm surgery. These aspects may prove to be crucial in improving control over the disease. Beliefs about internal pain control may prove to be a useful target for psychological interventions to improve personal pain control and to increase control over the disease and its course.

## Figures and Tables

**Table 1:** Pain-control beliefs.

*BPCQ*	*N*	*Min*	*Max*	*M*	*SD*
Internal control	42	7	29	21.02	4.96
Influence of doctors	42	10	24	19.09	3.9
Influence of chance	42	7	24	15.28	3.69

*N*—number of respondents; *M*—mean value; *SD*—standard deviation.

**Table 2:** Internal control vs. gender and type of surgery.

***Internal Control vs. Gender***	***N***	***M***	***SD***	***Z***	***p***
Woman	7	22.42	2.29	0.42	0.671
Man	35	20.74	5.31		
***Internal control vs. type of surgery***	***N***	***M***	***SD***	***Z***	***p***
Open surgery	28	20.64	5.79	0.06	0.947
EVAR	14	21.78	2.63		

EVAR—endovascular aneurysm repair; *N*—number of respondents; *M*—mean value; *SD*—standard deviation; *Z*—normal distribution, the result of the *Z* test; *p*—level of sig-nifi-cance.

**Table 3:** Internal control vs. type of aneurysm.

*Internal Control vs. Type of Aneurysm*	*N*	*M*	*SD*	*H*	*df*	*p*
Asymptomatic	24	21.37	4.39			
Symptomatic	15	22.06	4.21	5.26	2	0.036
Ruptured	3	17.92	7.21			

*N*—number of respondents; *M*—mean value; *SD*—standard deviation; *H*—unevenness coeffi-cient; *df*—degrees of freedom; *p*—level of significance

**Table 4:** Internal control vs. age, BMI, and nicotine addiction.

***Internal Control***	***N***	***rHO***	***p***
Age	42	−0.04	0.800
BMI	42	0.09	0.556
***Internal control***	***N***	***rHO***	***p***
Number of cigarettes smoked per day	42	−0.49	0.010

*N*—number of respondents; *rHO*—Spearman’s rank correlation coefficient; *p*—level of sig-nifi-cance.

**Table 5:** Influence of doctors vs. gender.

***Influence of Doctors vs. Gender***	***N***	***M***	***SD***	***Z***	***p***
Woman	7	2/* 0.42	5.44	1.59	0.110
Man	35	19.89	3.56		
***Influence of doctors vs. type of surgery***	***N***	***M***	***SD***	***Z***	***p***
Open surgery	28	18.57	4.08	1.18	4.08
EVAR	14	20.14	3.41		4.08

*N*—number of subjects; *M*—mean value; *SD*—standard deviation; *Z*—normal distribution, the result of the Z test; *p*—level of significance. *p* < 0.05.

**Table 6:** Influence of doctors vs. age, BMI, and nicotine addiction.

***The Influence of Doctors***	***N***	***rHO***	***p***
Age	42	0.38	0.013
BMI	42	0.14	0.386
***The influence of doctors***	***N***	***rHO***	***p***
Number of cigarettes smoked per day	42	−0.15	0.459

*N*—number of respondents, *rHO*—Spearman’s rank correlation coefficient, *p*—significance level.

**Table 7:** Influence of doctors vs. type of aneurysm.

*Influence of Doctors vs. Type of Aneurysm*	*N*	*M*	*SD*	*H*	*df*	*p*
Asymptomatic	24	19.33	4.1	0.58	2	0.748
Symptomatic	15	18.53	3.88			
Ruptured	3	20.01	3.02			

*N*—number of respondents; *M*—mean value; *SD*—standard deviation; *H*—unevenness coefficient; *df*—degrees of freedom; *p*—level of significance.

**Table 8:** Influence of chance vs. gender and type of surgery.

***Influence of Chance vs. Gender***	***N***	***M***	***SD***	***Z***	***p***
Woman	7	14.42	4.39	0.28	0.773
Man	35	15.45	3.59		
***Influence of chance vs. type of surgery***	***N***	***M***	***SD***	***Z***	***p***
Open surgery	28	15.57	3.82	0.32 0.748	
EVAR	14	14.71	3.49		

*N*—number of respondents; *M*—mean value; *SD*—standard deviation; *Z*—normal distribution, the result of the Z test; *p*—level of significance.

**Table 9:** Influence of chance vs. age, BMI, and nicotine addiction.

***Influence of Chance***	***N***	***rHO***	***p***
Age	42	−0.26	0.093
BMI	42	−0.13	0.416
***The influence of doctors***	***N***	***rHO***	***p***
Number of cigarettes smoked per day	42	0.50	0.009

*N*—number of respondents; *rHO*—Spearman’s rank correlation coefficient; *p*—level of significance.

**Table 10:** Influence of chance vs. type of aneurysm.

*Influence of Chance vs. Type of Aneurysm*	*N*	*M*	*SD*	*H*	*df*	*p*
Asymptomatic	24	14.95	3.78	1.6	2	0.449
Symptomatic	15	15.2	3.34			
Ruptured	3	15.96	3.26			

*N*—number of respondents; *M*—mean value; *SD*—standard deviation; *H*—unevenness coefficient; *df*—degrees of freedom; *p*—level of significance.

## Data Availability

A dataset will be made available upon request to the corresponding authors one year after the publication of this study. The request must include a statistical analysis plan.
